# Development of an educational intervention to reduce the burden of adult chronic lung disease in rural India: Inputs from a qualitative study

**DOI:** 10.1371/journal.pone.0254534

**Published:** 2021-07-15

**Authors:** Biswajit Paul, Rita Isaac, Hemalatha R., Paul Jebaraj, Muthathal S., Deepa Das, John Norrie, Liz Grant, Hilary Pinnock, Harish Nair, Aziz Sheikh, David Weller, RESPIRE Collaboration

**Affiliations:** 1 RUHSA Department, Christian Medical College, Vellore, India; 2 Usher Institute, The University of Edinburgh, Edinburgh, United Kingdom; 3 Pulmonology and Critical Care, Bangalore Baptist Hospital, Bengaluru, India; ESIC Medical College & PGIMSR, INDIA

## Abstract

**Background:**

Chronic respiratory diseases (CRDs) are major causes of mortality and morbidity worldwide with a substantial burden of the disease being borne by the low and middle income countries (LMICs). Interventions to change health behaviour which aim to improve the quality of life and reduce disease burden due to CRD require knowledge of the problem and factors influencing such behaviour. Our study sought to appreciate the lived experiences of people with CRD, their understanding of the disease and its risk factors, and usual practice of health behaviour in a rural low-literate community in southern India.

**Methods:**

Qualitative data were collected between September and December 2018 through eight focus group discussions (FGDs), five in-depth interviews and four key-informant interviews from patients and community members. Community engagement was undertaken prior to the study and all interviews and discussions were recorded with permission. Inductive coding was used to thematically analyse the results.

**Results:**

Major themes included understanding of chronic lung disease, health behaviours, lived experiences with the disease and social norms, attitudes and other factors influencing health behaviour.

**Discussion:**

Poor understanding of CRDs and their risk factors affect health seeking behaviour and/or health practices. Stigma associated with the disease and related health behaviours (e.g. inhaler use) creates emotional challenges and mental health problems, besides influencing health behaviour. However barriers can be circumvented by increasing community awareness; communication and connection with the community through community based health care providers can turn challenges into opportunities for better health care.

## Introduction

Chronic respiratory diseases (CRD) constitute a major and escalating challenge to public health in low-and middle-income countries (LMICs) because of their high prevalence and severity—and limited awareness of the risk factors and treatment options in the population, especially in the rural, low-health-literate communities [1–3]. Around 90% of the deaths from chronic obstructive pulmonary disease (COPD) occur in LMICs [4, 5]. COPD was the second most common cause of mortality and morbidity [in terms of disability adjusted life years (DALYs) lost] in India in 2016 [6]. COPD and asthma were responsible for 75·6% and 20·0% of the chronic respiratory disease DALYs, respectively, in India in 2016 [7].

Engaging with communities and health systems in CRD management is critical to improve quality of life of people affected. To that end, evidence-based control measures like smoking prevention, avoidance of exposure to biomass fuels, reduction in exposure to environmental allergens and better access to standardised protocol based management are recommended [1, 8–10]. It is challenging for health systems to adapt to meet the chronic long-term care needs of their populations related to CRDs while continuing to provide curative care services for acute diseases. With limited clinical and workforce resources, identifying the most effective interventions to reduce the risks, drivers, causes and impact of CRD, is important. We undertook a study on behavioural change using an educational intervention based on the Theory of Planned Behaviour (TPB), and explored the feasibility of front-line health care worker driven community education, screening, referral and treatment, with community follow-up [11]—an approach suitable for low-resource settings.

As a prelude to developing a more structured and systematic intervention study, it was necessary to understand the social, financial and cultural context of our participants and their living environment; in particular their lived experiences and attitudes toward CRD along with perceptions of caregivers and community perspectives about the disease. This was necessary for the development and implementation of an intervention which could address the gaps in beneficiary-provider cycle and make it effective in changing health behaviour. Therefore, we undertook a qualitative study to gain insights into individual and community perceptions, opinions, practices, attitudes, norms (cultural and subjective), beliefs and barriers to CRD prevention and care.

## Methods

### Ethics statement

Ethical approval for this study was granted by the scientific and ethics committee of Christian Medical College, Vellore, India known as the Institutional Review Board (IRB) of CMC Vellore vide IRB min no. 11381 dated 27.06.2018. and approval was granted from the Health Ministry’s Screening Committee of Indian Council of Medical Research with proposal id 2018–0706. This study is one part of a large mixed-method study which was approved by the Research Governance body of the University of Edinburgh. Written informed consent was obtained from all the participants who volunteered for the study.

The objectives of our qualitative study were guided by the theory of planned behaviour (TPB), wherein we wanted to know about the attitudes, social norms, perceptions and barriers to health behaviour among the patients of CRD and in the community. Our topic guide was constructed keeping these objectives in mind and elicitation of the lived experiences from the patients of CRD was a part of such inquiry. Guided by TPB, we had conducted interviews among caregivers and the key-informants to obtain information about societal barriers, perceptions of community about CRD and their opinions on related health behaviour. The narratives from the patients, caregivers and the key community members thus obtained were interpreted by thematic analysis framework to arrive at the results.

### Setting and study design

Our CRD patients were from a rural community development block (administrative unit) with a population of 128,000; they had low literacy levels and were from lower socio-economic classes. They utilised health care services provided by the Rural Unit for Health and Social Affairs (RUHSA), a secondary level hospital of Christian Medical College, Vellore located in the block, through its 18 outreach clinics (in 18 clusters) in the community providing primary care. This study was conducted from September to December 2018. The qualitative survey guide was developed from the five constructs of the theory of planned behaviour that included CRD related behaviour, behavioural intention, attitude, subjective norm and perceived behavioural control. We hypothesised that the five components of the TPB would better predict CRD-related behaviour.

This study used qualitative research methods to collect information. A total of eight focus group discussions (FGDs), involving 51 participants, five in-depth interviews (IDIs) and four key informant interviews (KIIs) were conducted–sufficient, through our pilot-testing, to achieve data saturation. FGDs were conducted among the patients with CRDs, IDIs with the care-givers and KIIs were with key community members.

### Community engagement, focus group discussions and the interviews

Prior to organising the focus group discussions and the interviews, community engagement was initiated with key community members including panchayat (local government) leaders, teachers, village leaders and self-help group members (small self-reliant, financially independent local women groups involved in income generation and community influencers) to apprise them about the CRD program and share their views and opinions towards it. It helped us in strengthening the relationship with the community and carrying out the study.

Participant selection: For the focus groups, initially, a sampling frame of all individuals with a diagnosis of CRD was developed from out-patient department registers of the secondary hospital and electronic database of outreach care services. Sampling techniques were refined through discussions with the health care workers, and patients were added based on their knowledge of patients living with CRD. Study participants were chosen purposively to represent the diversity within the study population in terms of age, gender and type of CRD. The inclusion criteria were adults above 18 years, both men and women who’d been diagnosed with CRD (including asthma, COPD or bronchiectasis) and resided permanently within the study region. The health care workers made home-visits to inform the potential participants about the study and to arrange a time and date for discussions. FGDs were conducted separately for men and women, stratified by age (<50 or ≥ 50 years) for homogeneity in group composition. Work environments and cultural practices of males were different from females, whereas the attitude and perceived control over a behaviour varied more by age, which made it pertinent to divide them into such groups. Written informed consent was obtained from the participants after they went through the information sheet with details of the study, in vernacular language.

Development of FGD guides: FGD guides were prepared based on themes resonating with the constructs of TPB; there were suitable prompts for symptoms of disease, risk factors and health behaviours, attitudes, social norms, the influence of peers, family members and the community and their belief in themselves and dependence on others for performing the behaviour. All these guides were prepared in English, translated to local vernacular language (Tamil) and back translated to English to retain its context and the meaning. It was piloted in a non-study village and the guides were refined for the definitive study.

A social scientist in our research team conducted the FGDs in the local language—assisted by an observer who took notes and captured interactions within and between participants and the moderator. Focus group discussions were conducted with the eight groups of patients in their respective villages at a place ensuring confidentiality. The focus groups with homogenous composition in terms of age, disease and gender and belonging to the same village helped the participants to volunteer opinions and attitudes about their disease experiences and provided us with enriching information about CRD perception and health behaviour.

The in-depth interviews (IDIs) explored the experiences of the caregivers and their views on caring and living with the patient with a CRD. The key informant interviews (KIIs) gathered community perceptions and opinions about CRD. The key informants had specific knowledge about and influence in the community. They were either local leaders or administrators in the community and comprised of block (administrative unit of local government) development officer, panchayat member, block medical officer and head of high school. They were chosen based on their experience and knowledge of the community and/or the health problem. The social scientist steered the IDIs whereas the key informant interviews were undertaken by the principal investigator (PI), a clinician with experience in CRDs. IDIs were conducted at home and KII at the workplace of participants.

All interactions were face-to-face. An audio-recorder was used to record the FGDs and the interviews. The length of the FGDs was 60–90 minutes while most of the interviews were about 30–45 minutes in duration.

### Analysis of qualitative data

The FGDs and interviews were transcribed in verbatim and translated into English. Analysis was conducted using a thematic analysis framework. An inductive coding frame was developed collaboratively between the investigators to allow the themes to emerge from the content of the raw data. This was done by reading the transcripts and also listening to audio. BP, RI and HR read through the transcripts to become familiar with the data. In doing so we were cognisant of our own perspectives on the problem, and while we are a multi-disciplinary group, our natural inclination was to impose a biomedical framework on the data. Being in a position of interacting with such patients on a daily basis, experience of working in the community for many years and closely observing the community where they come from and where this particular research was conducted, our inclination for biomedical terms was natural and reflected in development of some of the themes, as in, causation, health behaviour and treatment seeking. However we went about with an open mind to capture participants’ perspectives, experiences and emotions during the process of interviews and discussions. The process of reading of the transcripts was an enriching experience of delving into the participants’ world and understanding their views. The interpretation was obtained by looking into the viewpoints of different stakeholders in their context. Nevertheless, the final result was an outcome of an iterative process of reading and re-reading the transcripts and understanding it in the context of participants’ environment and own knowledge.

Initially data from two FGDs, one KII and IDI each were coded separately by BP and HR and quality checked by RI and DW. Data from the transcripts were charted into a matrix using Microsoft excel, adjacent to appropriate codes; any emerging codes were also added to the matrix. Subsequently themes and sub themes were developed by analysing the text and finding commonalities and differences within and between the codes. Descriptive accounts of the data were written by BP and RI based on the chart developed. As part of the analysis, similarities and differences were explored between the different data collection methods and different community members’ accounts. Such interrogation of the descriptive accounts was used to generate an explanatory account of the data. The final document, with the themes and the narration, was read and approved by all the authors.

## Results

The profile of the participants are illustrated in Tables 1 and 2.

**Table 1:** Focus group discussion participant characteristics.

Group composition	No. of participants)
Village 1 FGD 1 (Females<50 years)	7
Village 2 FGD 2 (Males≥50 years)	7
Village 5 FGD 3 (Females<50 years)	6
Village 6 FGD 4 (Males≥50 years)	6
Village 7 FGD 5 (Females≥50 years)	6
Village 8 FGD 6 (Males<50 years)	7
Village 3 FGD 7 (Females≥50 years)	6
Village 4 FGD 8 (Males<50 years)	6

**Table 2:** Participant characteristics of In-depth interviews and key informant interviews.

In-depth interviews (IDI) Caregiver gender and age	Gender & Age (Relationship to the patient)
IDI-1	Male, 59 years (Husband)
IDI-2	Male, 56 years (Husband)
IDI-3	Male, 39 years (Son)
IDI-4	Female, 31 years (Daughter)
IDI-5	Female, 62 years (Wife)
Key informant interviews (KII)	Gender, Age (Position)
KII-1	Male, 50 years (Block Development Officer)
KII-2	Male, 40 years (Block Medical Officer)
KII-3	Male, 45 years (Panchayat (local government) member)
KII-4	Female, 60 years (School Principal)

Based on the thematic analysis, our results have been divided into three major areas (themes) each encompassing sub themes. The first area evolving from the focus group discussions and the interviews with the key informants and care-givers focusses on “understanding of chronic lung disease and associated health behaviour”. It explores their perception of the disease condition and the health behaviour in practice. The second area stemming from the same data is based on participants’ ‘lived experiences with the disease’ and describes their health experiences and social issues arising from the disease. The third theme ‘social norms, attitude and other factors influencing health behaviour’ provides insights into why people do what they do, influenced by societal norms, and how these behaviours can be modulated to improve their health.

### Understanding of chronic lung disease and associated health behaviour

#### Perception about their disease condition and their causation

Participants generally understood their disease to involve difficulty in breathing or a feeling of breathlessness. They appreciated it was long term in duration and something that was difficult to manage. They described various terms like breathing difficulty/breathlessness (*muchi kastam–in local language*), or shortness of breath (*moochu prachnay/wheezing*) and asthma to describe their condition and also knew it stayed for long periods affecting their daily life:

*‘I have this wheezing (moochu prachnay) for 10 years’ (FGD4, R4); ‘From childhood days [I am] suffering from wheezing problem (moochu kahstam).’ (FGD8, R3)*

One participant mentioned that lungs were affected by the disease, as she had been told by a doctor. They knew that their disease was made worse by seasonal factors, exertion, dust, cold weather, and by smoking; they considered these to be causes of their disease. Some participants stated that sharing food with asthma patients led to asthma. Though participants commonly used biomass fuels for cooking, they did not realise that the smoke produced by its use was a potential risk factor and was associated with their respiratory condition.

*‘Doctor said [I have] asthma and it will affect my lungs.’ (R3, FGD 5) ‘Due to dust (tuci), rainy season (malai kalam), if it’s heavy rainfall disease will become severe…’ (R1, FGD1); ‘For me, sambirani smoke [is the] cause for this problem’. (R3; FGD8); ‘I got it from my mother-in-law because she gave the food, after eating with her from the same plate and also what she left in the plate…So I got asthma from her’. (R5; FGD5)*

*‘No [do not get the disease by cooking with firewood]. In olden days we used to cook in firewood only.’ (R1, FGD 3);*

The key community members opined that people described their disease in terms of their symptoms–but that general awareness levels about CRDs were low in the community.

*‘If we ask the people in the society, they will tell what problem they have, the symptoms like breathing difficulty (muchi kastam) but they do not know the name of the disease.’(KI-3)*

*‘Awareness level is very low, they don’t know. Parents who are coming for parents meeting will know because we are conducting some awareness program. All the people in the society may not aware of that [CRDs].’(KI-4)*

#### Treatment seeking for chronic respiratory disease

The local community commonly relied on western medicines (*Allopathy*) for treatment; however some of them used traditional forms of medicine (*Ayurveda*, *Siddha*) and local treatments with a belief that their disease could be cured.

*‘[Go for] only allopathy’. (R1, FGD2); “No, not going for other treatment, we all following only allopathy.’ (R6, FGD5); ‘Yes, I went to Siddha and Ayurveda, but I do not want that now, I like to continue Allopathy medicine only.’ (R1, FGD3)*

Patients sought treatment from a range of health care facilities and services including RUHSA hospital, government hospitals, local clinics and medical stores (pharmacies are known as medical stores in this region where medicines are stored and distributed with or without prescription). However it was a common practice to initially go to medical stores to get medicines for relief of symptoms. Regularity of treatment was based on how severe they perceived their symptoms to be, patients obtained medicines only at the time of the symptoms or seasonally; typically the local medical stores were first line of seeking treatment before they engaged with mainstream healthcare services (providing western medicine or allopathy). The key-informant interviews substantiated the above treatment seeking behaviour of the patients.

*‘Visit RUHSA and government hospital.’ (R3, FGD2); ‘Not using medicine regularly, only at winter season.’ (R5, FGD1); ‘No, not taking [treatment] continuously; whenever I had cough and cold I will buy medicine in the medical shop and use it. I will not go to hospital.’ (R6, FGD5)*

*‘Patients are not going to the hospital at their initial stage of the disease; after getting severe they go to hospital; At the beginning they will go to local doctors, it became severe they will visit RUHSA; …(go to) medical shops or local doctors in the beginning.’(KI 3)*

#### Health behaviour related to chronic respiratory disease

Male participants spoke openly about their smoking habits and appreciated that smoking was the possible reason for their disease. Both male and female respondents spoke about using biomass fuels (wood, crop residue, animal dung) for cooking their food as a common practice. They cited the higher price of gas cylinders and easy availability of biomass fuels as reasons for doing so. Cooking gas, though available, was used infrequently.

*‘Smoking causes the disease…smoking for more than five years; using firewood at home…don’t have gas facility.’ (R2, FGD 2); ‘[using firewood] because cost of cylinder is increased’ [Rs 1000 per cylinder;, 1$~ Rs73] (R1, FGD 5); ‘While going for coolie work, we will collect firewood. It’s free of cost, so we are using it.’ (R2, FGD 6)*

Participants described medications (*marunthu*–local language) used for treatment; tablets were often used (*mattirai–*local language) and injections (*uci*- local language) while nebulisation (*aavi*–local language) was only used when they went to hospital for increased breathlessness. Use of inhaler (*puff/pump–*local language) was infrequent, used only by a few and typically used for quick relief of symptoms.

*‘[I take] tablets (mattirai) provided by government hospital [for my breathing difficulty].’ (R2, FGD 2); ‘[I take] tablet (mattirai) and injection (uci) [for my respiratory problem].’ (R2, FGD 4)*

*‘Using inhaler (puff) is good, it gives sudden relief to me.’ (R1, FGD2); ‘I‘m satisfied about using puff, but only two minutes control.’ (R1, FGD8)*

The interviews produced similar reports about inhaler use—its use was infrequent but when used provided better symptom control.

*‘Before treatment she found difficult to face the problem of wheezing. After treatment [with inhaler] she feels better in handling the issue.’ (IDI 2)*

The participants of the FGDs were not aware of any breathing exercises that should be done for their respiratory condition; neither did they appreciate that exercises are helpful and provide better symptom control. They explained that they were not informed about breathing exercises by health providers.

*‘Not doing exercise, only taking medicine.’ (R1, FGD 1); ‘Nobody is doing exercise….nobody taught us exercise.’ (R3, FGD3);*

### Lived experiences with the disease

#### Health related experiences from CRD

Health experiences arising from CRD were typically reported by the participants as physically disabling and leading to the most ‘difficult moments’ of their lives; at times they felt helpless as they were unable to work, unable to sleep and even breathe normally. This was reiterated by the interviews from the caregivers of the CRD patients.

*‘I cannot work hard. Even for doing small and simple work I have to sit and take rest a while then only I can work.’ (R5, FGD 6); ‘[I have] chest pain while walking, felt difficulty in breathing; nobody should have this disease, when I had breathlessness it was very difficult for me… (started crying).’ (R4, FGD 1)*

*‘… she finds difficulty in breathing… doctor will give nebulisation (aavi) to the patient and will prescribe medicine. Then we will bring the patient to home and give medicines with hot water…If she eats something cold she will have severe breathing problems. So we provide her chapati, ginger tea…; During winter and rainy seasons, I won’t allow her to work out of the house because during winter she will suffer a lot.’ (IDI 4)*

#### Social interactions and experiences due to the disease

Participants’ experiences in their community due to their disease were often very uncomfortable, distressing and emotionally challenging. Because of their disease, they felt sad and depressed, they were often ill-treated and ‘looked down upon’—and they felt powerless to do anything about it. So distressing was the experience of living with the disease that participants often broke down while reporting it.

*‘…because he (doctor) revealed that I have asthma. I was shocked, didn’t expect from him…As soon as I heard about my illness I felt unhappy and depressed. (R4, FGD 5)*

*‘If I cook food, my relations and neighbours will hesitate to eat and if I give water they will not drink; those situations affect me very much; I looked after… cared my husband’s elder brother’s daughter… (pauses and starts speaking with a heavy voice). As soon as they came to know [about] my respiratory disease he will not allow her daughter to my house and stop talking to me. (while saying this she started crying).’ (R2, FGD 1)*

Taking care of a patient with CRD was also challenging, both physically and mentally; the caregivers typically shared the emotions along with the patient.

*‘If she is in treatment she will be ok. If she had relapse she will again get into wheezing problem. If she gets clear with her disease, I’m happy because she will take care of the family members and also all the family issues’ and affairs, my responsibility will be reduced; If she is psychologically good and happy I feel relaxed; I felt afraid, very bad and afraid whenever she had wheezing problem.’ (IDI 1)*

### Social norms, attitude and other factors influencing health behaviour

#### Family and community influence on health behaviour

Family played an important role in the life of each individual and most of the participants reported that their family would help them to get treatment, provide financial support and encourage them to take nutritious food and use inhalers. Social and psychological support of the patients by family members was also evident in the in-depth interviews with the caregivers.

*‘Family members encourage and take care,’ ‘[I am] taken to hospital [by family members].’(R6, FGD1) My wife advised me to eat all the fruits and vegetables, [she said] don’t ignore anything, nothing will happen to you.’ (R2, FGD 4)*

*‘…he is depending on me much. If he has wheezing, he is unable to talk and I will assist him and help him to inhale puff(inhaler); Mentally I’ll give full support to him not to became tensed. If sometime somebody will speak something which creates tension to him I’ll face the situation, not allow him to interfere,… We always give importance for the patient’s peacefulness.’ (IDI 5)*

Participants typically did not want to use inhalers in public and tried to hide their disease from neighbours/friends as they felt they would be stigmatised and ridiculed; they would prefer, rather, to keep their disease and medication use hidden from their communities or not take medications at all.

*‘Don’t know what the neighbours think…will use in my house, they might not know my problems. If I go for any function that time I will face difficulty.’ (R1, FGD 1) ‘I’m using inhaler (puff) because I’m using inside my house, I will not bring outside.’ (R2, FGD 1)*

*‘Not interested to reveal their problems in front of the others because of stigma. They will find difficult to share with others.’ (KI 4, Head Government Girls High School))*

*‘Many know the respiratory problem but they are not ready to use puff (openly)… that means in public.’ (KI 3, Panchayat member)*

#### Patient’s attitude towards disease and health behaviour

Participants expressed their desire to ‘get better of the disease’; they felt it was difficult and distressing to live with the disease. They expressed a desire to lead a healthy, independent life where and can take care of their family.

*‘[I] want to be cured [of this disease].’ (R1, FGD 2); ‘[I want] to take care of my health and to take care of my family members.’ (R3, FGD 4)*

Those who were using inhalers felt that using them was beneficial for them. The positive attitude towards inhalers was also often echoed by caregivers of CRD patients on inhalers:

*‘It is useful…feeling very good [after using inhaler]’. (R2, FGD5)*

*‘To get permanent solution, she is using inhaler (puff);…to keep herself healthy.’ (IDI 3)*

#### Other societal influences

There were some barriers to positive health behaviours–relating to money and time, or social and health system limitations:

*‘Both time and money is a major factor. Sometimes there is nobody to accompany to go to the hospital.’ (R5, FGD 7) ‘Yes [I use puff], but not using now because, I don’t have sufficient money to buy inhaler [puff].’ (R1, FGD 6)*

*‘If she needs to attend funeral ceremony (few important unavoidable occasions) cannot continue treatment at that time, do not want others to know about it.’ (IDI 4)*

*‘They are not following due to family problem, some people have job problem. Some people are smokers, drinkers, we cannot believe and cannot expect they take treatment regularly; They need good treatment, in government side there is no supply of inhalers: …need to get permission from higher officials…’ (KI 2, BMO)*

Key informants highlighted positive government initiatives, such as giving gas cylinders at subsidised rates, people getting financial support through work and discouraging open burning of waste:

*‘Government has given gas connection; They are earning 200 to 300 rupees per day (through 100 days’ work under MGNREGA) and they can buy gas (at subsidised rates)…They (waste collectors) will collect the waste things at home in the morning at 7.O clock…every day… instead of burning should be given to them; They collect and segregate at one particular place and put in the separate pit.’ (KI 1, BDO)*

Subsidised treatment, establishing local community clinics, starting a separate respiratory care unit for CRD patients, and providing psychological support were some suggestions by the participants to improve lives and health behaviour.

*‘if I get concession in treatment and investigation that will nice.’ (R2, FGD1) ‘free medicines and organise medical camp once in a month at the panchayat (local community)’. (R1, FGD 8)…(others nod their head in agreement)*

*‘To give separate care and start unit for respiratory problem.’ (KI 3, Panchayat Leader)*

*‘…so, the wheezing problem is very serious disease, so you should arrange counsellors for counselling the patient for regular treatment and follow up.’ (IDI 1)*

## Discussion

### Principal findings, and strengths and limitations of the study

#### Principal findings

CRD patients in this study had gaps in understanding of CRD causation, risk factors and long-term implications. This is likely to have contributed to continued health behaviours including use of biomass fuels, delayed treatment-seeking, irregular and infrequent use of inhalers. Other factors, such as financial constraints limiting gas use in cooking and lack of communication regarding breathing exercises from health providers were unrelated to awareness or understanding–respondents’ circumstances dictated health behaviour choices. Social norms and cultural barriers to using inhalers in public, disclosure of their disease, and use of traditional remedies further restricted desired health behaviours. Their experience of health-related issues with the disease, stigma and social exclusion led to psychological disturbance and depression, while negatively influencing their health behaviour. Nevertheless, they typically had family support and a positive attitude towards their health.

#### Strengths and limitations of the study

The strengths of the study were the richness and validity of the data, enabled by our triangulation [12] of findings achieved by using different methods of data collection, coding and quality checks by the team and obtaining information from different groups of participants. Providing a rich account of data and its confirmability also added to its quality. Both the gender and age groups were adequately represented to obtain a diverse sample of people to describe their experiences. The sampling in the FGDs involving community members without disease could have provided a richer narrative of community perspective of the CRD in addition to the information obtained from KIIs. Interviews with different levels of health care providers could have provided more information about health system barriers, which in our study was limited to only one health provider. There may have been some social desirability bias [13] operating during the FGDs and KIIs, due to the typically long periods of health service in the community and good rapport thereof, but it would be limited to specific questions and individuals. The discussions and the interviews were led by a social scientist with experience in qualitative research and interpretation included discussions with the multidisciplinary team.

### Interpretation in the light of published literature

This paper highlights poor awareness about CRD among rural low literate population and their associated health behaviours–placed in the context of their social-cultural milieu, the health and social consequences of the disease and the barriers and facilitators to health behaviour.

For a population to adopt health behaviours which prevent chronic disease and ensure adequate management, it is essential to have a understanding of the disease, its associated risk factors and treatment options and awareness of the benefits and outcomes of their health behaviours. Healthy behaviours are feasible–financial, social or cultural constraints may limit choice. CRD awareness levels in this rural community were limited to symptoms, duration, seasonality and available treatments for exacerbations of symptoms (whether or not they worked). There was poor understanding of the condition, causes and risk factors for the disease with prevailing misconceptions—like spread of the disease from one person to another through food or contact. Respondents were typically unaware that their disease was not curable, and didn’t appreciate it could be well controlled by regular medications, especially uninterrupted use of inhalers for maximum benefits.

Our findings resonate with those from similar studies; a qualitative study on patients’ experiences of living with chronic respiratory disease in Uganda found low awareness levels and lack of knowledge about the condition with an unfulfilled expectation of being cured and misunderstandings among the family members and community [14]. Description of symptoms without knowledge of the disease condition has been reported in a study from Malawi [15]. This is in contrast to findings from the high income countries where patients typically have a good understanding of their disease, its severity, the system involved and the prognosis [16, 17]. Low awareness levels and understanding of the disease affect health seeking behaviour and/or health practices; this is often emphasised by theoretical models such as the Hierarchy of Effects Model (HOEM) which proposes that ‘proximal’ variables like awareness are causally linked to ‘distal’ variables like behaviour change through a series of intermediate measures, including social norms, attitudes and intentions [18, 19]. Similarly psychological theory-based models like the Health Belief Model (HBM) and the Integrated Behaviour Model (IBM) which incorporates the Theory of Planned Behaviour (TPB) also emphasise knowledge as a key determinant of health behaviour change [20].

The CRD related health behaviours we observed in our study participants were an outcome of a combination of factors–limited knowledge about the disease and its long term implications, prevailing social norms and cultural practices, financial and health system constraints, stigma associated with the disease and its psychological impact and disease-related social marginalisation. Biomass fuel was commonly used for cooking in the rural community; biomass fuel being one of the known indoor air pollutants [21, 22] and a common risk factor for CRD in LMIC settings [1, 22]. It was quite surprising to find that most of the participants were unaware of it as a risk factor for their disease and were happy to continue using it, even arguing in its favour as a long followed cultural practice. Nevertheless, some respondents highlighted cost as a reason to continue with these cooking methods–in poor settings, awareness of risk factors is often insufficient to avoid them. Other studies in LMIC settings have demonstrated limited awareness of the relation between smoke and respiratory health, contributing to extensive exposure to mostly biomass-related smoke [23].

We observed the common practice of attending nearby medical stores or get some local treatment for temporary relief of symptoms without consulting a doctor or going to a hospital practising western medicine. Participants also followed home remedies like avoiding certain types of food, and often sought the help of traditional systems of medicine, initially, with a belief of cure from the disease altogether. The delay in accessing care from mainstream healthcare services can result in disease progression, poor quality of life and additional economic burden on the family and the health system [24, 25]. Improving health literacy and understanding of the disease can lead to early health seeking from mainstream health care system [15]. Also the role of health care providers (HCPs) in communicating with patients about diagnosis, treatment and the course of the disease is extremely important [26–28].

The use of inhalers among the CRD patients was infrequent and irregular. Social norms influenced a person’s intention and perceived control to perform the behaviour–for example, although people knew inhalers provided quick relief of symptoms, they were reluctant to use them in public as they felt their communities did not approve of such behaviour which would result in social marginalisation. Previous studies have reported that patients with asthma felt embarrassed while using inhalers in public [29]. Family and community norms reflect the social and environmental context within which an individual’s behaviour takes place, as described by the Social Ecological Model by McLeroy et al., 1988 [19, 30]. Individual health behaviours are influenced by perceived approval, or disapproval, of the behaviour by significant others (family or community), known as societal norms as per Theory of Planned Behaviour [20]. People who have good family support and are encouraged to perform the behaviour are more likely to do it—as explained by Ajzen’s Theory of Planned Behaviour [31]. Also the accessibility and availability of modern health systems and medical devices (coupled with their affordability) are other factors which can act as deterrent to appropriate health behaviour. Study participants emphasised the lack of availability of inhalers at government health centres—and they sometimes could not afford to buy them from pharmacies.

One of the surprising findings of our study was the limited knowledge or practice of breathing exercises by the participants, mainly due to limited information provided to them by heath care providers. Breathing exercises include different breathing techniques (e.g. pursed lip breathing) which can improve functional exercise capacity, ventilation and physical component of quality of life [32, 33]. Exercise is one of the vital components of pulmonary rehabilitation, presently considered to be the most accepted method of non-pharmacological treatment for improving the quality of life and functional capacity in patients with COPD and other CRDs [34, 35]. Therefore investing in pulmonary rehabilitation and breathing exercises, may help improve the quality of life of patients with CRDs. Also, better communication by health providers to patients about the breathing exercises and their benefits can prove invaluable in promoting such health behaviour.

The lived experiences reported by the participants suggested that CRD not only affected them physically but also caused psychological distress. It was not just another disease–it caused social seclusion and stigma in the community and led to depression. This disrupted their lives and their health behaviour. Such types of lived experiences were also reported by other studies among CRD patients, be it in the LMICs [15, 17, 30, 36–38] or in the developed world [39–42]. These experiences contributed to personal barriers to seeking treatment and adherence to treatment and hence overall physical and mental health outcomes [43, 44].

The positive elements which could be pivotal in changing health behaviour among these CRD patients were family support and the positive attitude of the patients towards regaining health. Family plays a big role in providing support–physically, economically and psychologically and therefore can be utilised to modulate patient’s health behaviour. However, changes in individual knowledge, awareness and attitudes would need to be accompanied by structural changes, for example–gas connections by the government at a subsidised rates that could reduce dependence on biomass fuel. Self-assured individuals using inhalers could act as catalyst of change to motivate others. Suggestions coming from the participants showed the importance was community engagement–solutions being grounded in the community where they lived with the problem. Implementing them may strengthen the health system and act as positive influences to respiratory health behaviour.

### Implications for policy and practice

A change at the policy level towards CRD focussing on investment to address financial barriers, education and health awareness, as well as implementation and availability of inhalers for use at primary care level can lead to significant change in perception of CRD in the community and behavioural practices among patients. These are often considered as ‘upstream’ factors or factors at the base of “the health impact pyramid” [45] which are difficult to achieve and require political will and government policy. They are, nevertheless, most effective and ultimately reach broader segments of society. Concerted efforts at the level of primary care practices and a better communication between patients and the HCPs can allay fears and misconceptions towards the disease, provide precise health information and promote healthy behaviour. Engaging the community with culturally acceptable and locally available media and methods, improving their awareness and acceptance of CRD prevention and treatment through educational interventions, building trust by improving interactions with the community through community-based health providers can result in immediate impact and outcomes.

This study helped us in identifying gaps wherein intervention could be useful, helping us in its design and delivery. Our intervention included culturally acceptable and easy to understand media and methods for improving awareness about CRD and related health behaviour. This was not limited to patients alone but was for dissemination in the community and the caregivers to help reduce stigma and generate social support. Detailed information about health practices related to CRD was given to the patients by the health care providers to plug the gap of limited or misinformation about behaviour practices; this was reinforced by training and demonstrations of health practices (inhaler use and breathing exercises). Inhalers were made available to all participating in the intervention and the community was engaged throughout the intervention period with appropriate health messages being delivered. Belief in themselves to perform health behaviour, along with community support might contribute to better health outcomes, thereby improving quality of life among the patients of CRD.

## Conclusions

Our study confirms that CRD-related attitudes and behaviours are influenced by knowledge and awareness–but also by a range of financial, social and other constraints. Even modest improvements in CRD preventive and treatment practices could have a significant impact on the quality of life of these patients. Health behaviours of CRD patients are rooted in their cultural practices and prevailing social norms, and influenced by lack of awareness of the disease and its long term consequences. Social marginalisation, stigma and psychological effects of the disease further impact their health behaviour. In a low resource setting of a LMIC among a rural low literate population, interplay of all the above factors modulate health behaviour. Therefore interventions are required at multiple levels to change behavioural practices related to CRD. At the individual (patient) level, raising awareness about the disease and making them engage with the modern health care system; at the community level, changing perceptions towards CRD and destigmatising the disease; at the health system level, better communication with patients and community engagement through frontline health care workers; and, finally at the policy level, there’s a need to address shortcomings in the beneficiary-provider cycle–at present, strategies and investment aren’t acknowledging the complexity of this disease and its behaviour and other determinants. There’s an urgent need to invest in strategies which will reduce barriers to healthy CRD behaviours, and to develop a CRD-specific health policy with evidence-based management protocols, supported by regular availability of and accessibility to medical equipment and medications.

## Supporting information

S1 Appendix Development of codes, sub-themes and themes. (DOCX)

S2 Appendix Qualitative topic guide. (DOCX)

S1 File Complete list of group authors of “RESPIRE Collaboration” for the manuscript. (DOCX)

## References

[1] Salvi S, Agrawal A. India needs a national COPD prevention and control programme. J Assoc Physicians India 2012;60 (Suppl.):5–7; 23155805

[2] McKay AJ, Mahesh PA, Fordham JZ, Majeed A. Prevalence of COPD in India: A systematic review. Prim Care Respir J. 2012;21:313–21; Vijayan V. K. (2013). Chronic obstructive pulmonary disease. Indian J. Med. Res. 137, 251–269 doi: 10.4104/pcrj.2012.00055 22790612 PMC6547954

[3] van Gemert F, Kirenga B, Chavannes N, Kamya M, Luzige S, Musinguzi P, et al. Prevalence of chronic obstructive pulmonary disease and associated risk factors in Uganda (FRESH AIR Uganda): a prospective cross-sectional observational study. Lancet Glob Health. 2015 Jan;3(1):e44–51. doi: 10.1016/S2214-109X(14)70337-7 . 25539969

[4] World Health Organisation. Chronic obstructive pulmonary disease (COPD) Fact sheet: World Health Organization; 2020. Available from: http://www.who.int/mediacentre/factsheets/fs315/en/.

[5] Salvi SS, Manap R, Beasley R. Understanding the true burden of COPD: the epidemiological challenges. Prim Care Respir J. 2012; 21:249–51. doi: 10.4104/pcrj.2012.00082 22885564 PMC6547972

[6] India State-Level Disease Burden Initiative Collaborators. Nations within a nation: variations in epidemiological transition across the states of India, 1990–2016 in the Global Burden of Disease Study Lancet, 390 (2017), pp. 2437–2460 10.1016/S0140-6736(17)32804-0 PMC5720596 29150201

[7] India State-Level Disease Burden Initiative CRD Collaborators. The burden of chronic respiratory diseases and their heterogeneity across the states of India: the Global Burden of Disease Study 1990–2016 Lancet Glob Health, 6 (2018), pp. e1363-e1374 10.1016/S2214-109X(18)30409-1 PMC6227385 30219316

[8] Lim MS, Lee CH, Sim S, et al. Physical activity, sedentary habits, sleep, and obesity are associated with asthma, allergic rhinitis, and atopic dermatitis in Korean adolescents. Yonsei Med J 2017; 58: 1040–1046. doi: 10.3349/ymj.2017.58.5.1040 28792151 PMC5552632

[9] Kaplan A, Thomas M. Screening for COPD: the gap between logic and evidence. Eur Respir Rev 2017; 26: 160113. doi: 10.1183/16000617.0113-2016 28298389 PMC9489098

[10] Ambrosino N, Bertella E. Life style interventions in prevention and comprehensive management of COPD. Breathe. 2018; 14(3): 186–94. doi: 10.1183/20734735.018618 30186516 PMC6118879

[11] Balasubramanya B, Isaac R, Philip S, et al. Task shifting to frontline community health workers for improved diabetes care in low-resource settings in India: a phase II non-randomized controlled clinical trial. Journal of Global Health Reports. 2020;4:e2020097. doi: 10.29392/001c.17609

[12] Korstjens Irene & Moser Albine (2018) Series: Practical guidance to qualitative research. Part 4: Trustworthiness and publishing, European Journal of General Practice, 24:1, 120–124, doi: 10.1080/13814788.2017.1375092 29202616 PMC8816392

[13] Paulhus DL. Two-component models of socially desirable responding. J Pers Soc Psychol. 1984; 46:598.

[14] Jones R, Muyinda H, Nyakoojo G, et al. Does pulmonary rehabilitation alter patients’ experiences of living with chronic respiratory disease? A qualitative study. Int J Chron Obstruct Pulmon Dis 2018; 13: 2375–2385 doi: 10.2147/COPD.S165623 30122917 PMC6087019

[15] Saleh S, Bongololo G, Banda H, Thomson R, Stenberg B, Squire B, et al. Health seeking for chronic lung disease in Central Malawi: adapting existing models using insights from a qualitative study. PLoS One. 2018;13(12):e0208188. doi: 10.1371/journal.pone.0208188 30557307 PMC6296555

[16] Schroedl CJ, Yount SE, Szmuilowicz E, Hutchison PJ, Rosenberg SR, Kalhan R.A qualitative study of unmet healthcare needs in chronic obstructive pulmonary disease. A potential role for specialist palliative care? Ann AmThorac Soc. 2014;11(9):1433–8. doi: 10.1513/AnnalsATS.201404-155BC 25302521 PMC4298987

[17] Jones RCM, Hyland ME, Hanney K, et al. A qualitative study of compliance with medication and lifestyle modification in Chronic Obstructive Pulmonary Disease (COPD) Primary Care Respir J. 2004;13:149–54. 10.1016/j.pcrj.2004.05.006 PMC6750686 16701658

[18] Cavill N., Bauman A., 2004. Changing the way people think about health-enhancing physical activity: do mass media campaigns have a role? J. Sports Sci. 22 (8), 771–790. doi: 10.1080/02640410410001712467 15370487

[19] Kite J, Gale J, Grunseit A, Li V, Bellew W, Bauman A (2018) From awareness to behaviour: testing a hierarchy of effects model on the Australian Make Healthy Normal campaign using mediation analysis. Prev Med Rep 12:140–147 doi: 10.1016/j.pmedr.2018.09.003 30258762 PMC6152809

[20] Glanz K, Lewis FM, Rimer BK, eds. 2008. Health Behavior and Health Education: Theory, Research and Practice. San Francisco, CA: Jossey-Bass.4th ed. pp 49–50,77–78, 70–76

[21] R7: Jiang XQ, Mei XD, Feng D. Air pollution and chronic airway diseases: what should people know and do? J Thorac Dis. 2016 Jan;8(1):E31–40. doi: 10.3978/j.issn.2072-1439.2015.11.50 ; PMCID: PMC4740163. 26904251 PMC4740163

[22] Kurmi OP, Lam KB, Ayres JG. Indoor air pollution and the lung in low- and medium-income countries. Eur Respir J. 2012 Jul;40(1):239–54. doi: 10.1183/09031936.00190211 Epub 2012 Feb 23. . 22362845

[23] van Gemert F, Chavannes N, Nabadda N, et al.Impact of chronic respiratory symptoms in a rural area of sub-Saharan Africa: an in-depth qualitative study in the Masindi district of Uganda. Prim Care Respir J, 22 (2013), pp. 300–305 doi: 10.4104/pcrj.2013.00064 23817677 PMC6442820

[24] Larsson K, Janson C, Ställberg B, Lisspers K, Olsson P, Kostikas K, et al. Impact of COPD diagnosis timing on clinical and economic outcomes: the ARCTIC observational cohort study. Int J Chron Obstruct Pulmon Dis. 2019 May 13;14:995–1008. doi: 10.2147/COPD.S195382 ; PMCID: PMC6526023. 31190785 PMC6526023

[25] World Health Organisation. Chronic Respiratory diseases. Assessed online on 21st December, 2020 from https://www.who.int/gard/publications/chronic_respiratory_diseases.pdf.

[26] Tarn DM, Heritage J, Paterniti DA, Hays RD, Kravitz RL, Wenger NS. Physician communication when prescribing new medications. Arch Intern Med 2006. Sep;166(17):1855–1862 doi: 10.1001/archinte.166.17.1855 [PubMed] [CrossRef] [Google Scholar] 17000942

[27] Osterberg L, Blaschke T. Adherence to medication. N Engl J Med 2005. Aug;353(5):487–497 doi: 10.1056/NEJMra050100 [PubMed] [CrossRef] [Google Scholar] 16079372

[28] Davis MS. Variations in patients’ compliance with doctors’ advice: an empirical analysis of patterns o communication. Am J Public Health Nations Health. 1968 Feb;58(2):274–88. doi: 10.2105/ajph.58.2.274 ; PMCID: PMC1228155. 5688773 PMC1228155

[29] Snadden D and Brown JB. Asthma and stigma. Fam Pract 1991; 8: 329–335. doi: 10.1093/fampra/8.4.329 1800195

[30] Castelino F, Prabhu M, Pai MS, Kamath A. Lived experiences of patients with chronic obstructive pulmonary diseases (COPD)—qualitative review. Indian Journal of Public Health 2018;9:263. [accessed Jun 15 2020].

[31] Ajzen I., 1991. The theory of planned behavior. Organ. Behav. Hum. Decis. Process. 50 (2), 179–211.

[32] Holland AE, Hill CJ, Jones AY, McDonald CF. Breathing exercises for chronic obstructive pulmonary disease. Cochrane Database of Systematic Reviews 2012, Issue 10. Art. No.: CD008250. doi: 10.1002/14651858.CD008250.pub2 Accessed 08 March 2021. 23076942 PMC11371308

[33] Ubolnuar N, Tantisuwat A, Thaveeratitham P, Lertmaharit S, Kruapanich C, Mathiyakom W. Effects of Breathing Exercises in Patients With Chronic Obstructive Pulmonary Disease: Systematic Review and Meta-Analysis. Ann Rehabil Med. 2019 Aug;43(4):509–523. doi: 10.5535/arm.2019.43.4.509 Epub 2019 Aug 31. ; PMCID: PMC6734022. 31499605 PMC6734022

[34] McCarthy B, Casey D, Devane D, Murphy K, Murphy E, Lacasse Y. Pulmonary rehabilitation for chronic obstructive pulmonary disease. Cochrane Database of Systematic Reviews 2015, Issue 2. Art. No.: CD003793. doi: 10.1002/14651858.CD003793.pub3 Accessed 14 January 2021. 25705944 PMC10008021

[35] Pesut D, Ciobanu L, Nagorni-Obradovic L. Pulmonary rehabilitation in chronic respiratory diseases—from goals to outcomes. Pneumologia. 2008 Apr-Jun;57(2):65–9. . 18822868

[36] Gupta A, Ravaliya V, Mishra D, Dani V, Sodawala C, Shah H, et al. Assessment of knowledge, attitude, and behavior about the disease process and physiotherapy management in patients with chronic obstructive pulmonary disease: A qualitative study. J Educ Health Promot. 2019 Jan 29;8:15. doi: 10.4103/jehp.jehp_209_18 ; PMCID: PMC6378829. 30815486 PMC6378829

[37] Dua R.; Das A.; Kumar A.; Kumar S.; Mishra M.; Sharma K. Association of comorbid anxiety and depression with chronic obstructive pulmonary disease. Lung India 2018, 35, 31–36. [Google Scholar] [CrossRef] doi: 10.4103/lungindia.lungindia_537_16 29319031 PMC5760864

[38] Wong SY, Woo J, Lynn HS, Leung J, Tang YN, Leung PC. Risk of depression in patients with chronic respiratory diseases: results from two large cohort studies in Chinese elderly from Hong Kong. Int J Geriatr Psychiatry. 2006;21(3):233–238. doi: 10.1002/gps.1447 16440404

[39] Steindal S. A., Österlind J., Halvorsen K., Schjelderup T., Kive E., Sorby e L. W., et al. (2017). A qualitative study of women’s experiences of living with COPD. Nursing open, 4 (4), 200–208. doi: 10.1002/nop2.86 29085646 PMC5653384

[40] Hanneke van der Meide PhD, Truus Teunissen PhD, Leo H. Visser PhD, Professor MS and Ethics of Care, Merel Visse PhD. Trapped in my lungs and fighting a losing battle. A phenomenological study of patients living with chronic obstructive and pulmonary disease. Scand J Caring Sci; 2020; 34: 118– 127 doi: 10.1111/scs.12713 31099083 PMC7074040

[41] Yohannes AM, Alexopoulos GS. Depression and anxiety in patients with COPD. Eur Respir Rev. 2014 Sep;23(133):345–9. doi: 10.1183/09059180.00007813 ; PMCID: PMC4523084. 25176970 PMC4523084

[42] Johnson J. L., Campbell A. C., Bowers M. & Nichol A.-M. Understanding the social consequences of chronic obstructive pulmonary disease. Proc. Am. Thorac. Soc. 4, 680–682 (2007). doi: 10.1513/pats.200706-084SD 18073402

[43] Rose S, Paul C, Boyes A, Kelly B, Roach D. Stigma-related experiences in non-communicable respiratory diseases: A systematic review. Chron Respir Dis. 2017 Aug;14(3):199–216. doi: 10.1177/1479972316680847 Epub 2017 Jan 23. ; PMCID: PMC5720230. 28111991 PMC5720230

[44] Hatzenbuehler ML, Phelan JC and Link BG. Stigma as a fundamental cause of population health inequalities. Am J Public Health 2013; 103: 813–821. doi: 10.2105/AJPH.2012.301069 23488505 PMC3682466

[45] Frieden TR. A framework for public health action: the health impact pyramid. Am J Public Health 2010; 100:590–595. doi: 10.2105/AJPH.2009.185652 20167880 PMC2836340

