# Supplementary material for: Development of an educational intervention to reduce the burden of adult chronic lung disease in rural India: Inputs from a qualitative study
Source: PLoS One. 2021 Jul 15;16(7):e0254534. doi: 10.1371/journal.pone.0254534 (PMC8281992; doi:10.1371/journal.pone.0254534)
Supplement: S1 Appendix — (DOCX) [file pone.0254534.s001.docx]

**Appendix 1: Development of codes, subthemes and themes**

**Codes Subthemes Themes**

description of the disease

*Perception about the disease condition and causation*

duration of the disease

cause of the disease

risk factors of CRD

control/cure for the disease

***Understanding about chronic lung disease and practice of health behaviour***

*Treatment seeking for chronic respiratory disease*

types of treatment used

availing treatment from

regularity of their treatment

risk related health behaviour

*Health behaviour (HB) related to chronic respiratory disease*

inhaler use experience

practice of respiratory health exercises

tests related to their disease

health problems faced due to CRD *Health related experiences*

***Lived experiences with the disease***

social status and social interactions due to their disease

*Social interactions and experiences*

feelings and opinions on living with the disease

family support

stigma due to disease

*Family and community influence on HB*

stigma of inhaler use

***Social Norms, attitude and other factors influencing health* *behaviour***

community opinion

attitude towards health and disease

*Attitude towards disease and HB*

attitude towards inhaler use

barriers to regular treatment

*Other societal influencers*

barriers to inhaler use

suggestions to facilitate health behaviour
