# Supplementary material for: Development of an educational intervention to reduce the burden of adult chronic lung disease in rural India: Inputs from a qualitative study
Source: PLoS One. 2021 Jul 15;16(7):e0254534. doi: 10.1371/journal.pone.0254534 (PMC8281992; doi:10.1371/journal.pone.0254534)
Supplement: S2 Appendix — (DOCX) [file pone.0254534.s002.docx]

**Appendix 2: Qualitative topic guide**

**Focus Group Discussion**

OBJECTIVE

To explore the patients’ CRD-related knowledge, attitudes, subjective norms, perceived behavioural control, and behavioural intentions in order to develop a quantitative structured survey questionnaire for baseline and post intervention assessment and a TPB based educational intervention programme in a predominantly low socio-economic setting

INTRODUCTION SCRIPT: To be read by researcher/group facilitator:

You have been invited to take part in this research study because you have received care for asthma/lung diseases. Our study is looking at your experience of living with the disease, perceptions regarding the chronic lung disease and management and how to make your health care better.

For this discussion group I will invite you to share as much or little as you feel comfortable sharing with the other patients in this group. This conversation will be tape-recorded for only the researchers to hear. Any recording of you taken today will not be used without your expressed consent.

Thanks.

QUESTIONS FOR DISCUSSION:

Please introduce yourself and tell us how long you have had asthma/COPD/other lung disease. Thinking about your experiences as a chronic lung disease patient, what have been your perceptions, beliefs about your disease, what do you think caused it, what factors facilitated to take treatment, what were the barriers to treatment, what were the opinions of the other family members, neighbours about your disease, how has it affected your life.

A. Chronic Respiratory Disease

- Perception about and attitude towards the disease
- Health practices followed

1. What type of lung disease you have? (List the name of the disease)
2. What do you think caused your disease?
3. What are the risk factors for your diseases?
4. Do you think your disease can be cured/controlled?
5. What are the difficulties you face because of your disease?
6. What type of treatment you are taking for your disease?
7. a. Are you on regular medications or on intermittent medications as and when you are symptomatic?

b. How have you been advice by your doctor?

1. Are you satisfied with the treatment from one centre, or have you sought treatment from many centres and different system of medicine? (Discuss the experience of treatment taken)
2. Have you been advised to use inhalers/rota halers (puffs) for your disease? Are you taking it? How many of you did not take though advised and why?
3. Have you been advised about chest physiotherapy/exercise to improve your respiratory health? Do you practice it? And how often? If no, why?
4. What tests (investigations) have you done for your disease? What was the need and outcome?

B. Health related behaviour

- Attitude towards the health-related behaviour
- Societal and subjective norms about the health behaviour
- Perceived control over the health behaviour
- Smoking/biomass fuel use in cooking/dust in the environment

-Do you think smoking/biomass fuel use /dust/has caused your illness?

-If you are a current smoker, has anyone asked you to stop smoking to improve your health condition?

-why have you not stopped smoking? Sharing of experience (M)

-If you are still using biomass fuel, why haven’t you stopped using? list the reasons (F)

-if they are practicing indoor cooking using firewood, why haven’t they changed? (F)

-If their occupation involves working in the cloud of dust/quarry dust/smoke, why couldn’t they change the job/use mask or other protective measures

-What is your opinion about burning waste in the villages related to CRD?

- Do you take the treatment as directed by one physician or take opinion from many practitioners? If many, why?
- What is your opinion about using inhalers/puffs/rotahalers?

-Opinion about inhalers ; what family and neighbours think about inhalers

-able to use the inhalers by themselves or not ?

-Do you use them as directed by your doctor or not ? What is your experience after using inhaler? positive and negative Experience (good/bad, beneficial/not beneficial, decreases respiratory symptoms/no effect, waste of money/improves quality of life)

- What do you all think about doing regular /daily respiratory exercises?
- Do your family support/encourage going to hospital on scheduled appointment?
- Has the most important person in your life encouraged/discouraged you taking regular medications/ inhalers/doing exercises/ stop smoking…behaviour?
- Which factors will help you to complete the course of treatment recommended by the physician? Also factors that hinder completing the course?
- How can you overcome the hindering factors?
- Do you feel capable enough to follow the treatment instructions given by your doctor/nurse/physiotherapist?

C. Beliefs

- Behavioural beliefs
- Normative beliefs
- Control beliefs
- do you think your disease is caused by smoking/biomass fuel use/too much dust in the work area?
- What do you prefer to use? (oral medicines/inhalers)? Why?
- Do you believe your spouse/children/relatives want you to take treatment on long term basis/life long?
- If RUHSA or other health system plan a pulmonary rehabilitation programme including exercise training, education and self-management intervention …. Will you be happy to participate? if no reasons…
- Do you think there is stigma associated with inhaler use?
- Do you believe/think the society will be supportive if you use inhalers?
- Do you think believe you can overcome the constraints/problems associated with regular treatment or use of prescribed medicines/inhalers?
- Will you be able to continue the chest exercises?

D. Facilitators and barriers to health behaviour (Adherence/Health seeking behaviour)

(education, skills, habit, environmental constraints, salience)

- Factors promoting positive Health Behaviour
- Factors preventing positive Health Behaviour
- What will help you in improving your respiratory health?
- If you know that you have to continue treatment for your disease lifelong (which can help in controlling the symptoms and preventing complications),

1. what are the factors which will help you in doing so?

(discuss need for health education, skills training for inhaler use, forming habit of taking daily medicine or using inhaler)

ii) what will prevent you from doing so?

- (discuss about availability, cost factor, distance from health centre etc.)

E. Intention to perform health behaviour

- Will you use the inhalers/rota halers/nebulization if advised by your doctor?
- How long will you use it? (only during symptoms / 3 months winter / lifelong)
- Will you perform respiratory exercises (To improve your breathlessness) if as advised to you?
- How long will you continue to do it?
- Will you come to follow up treatment as advised by your doctor?
- How do you think do you have to use medicines/inhalers/respiratory exercises?

F. Improving Health Behaviour

- What are your suggestions for maintaining positive health behaviour (related to CRD)?
- How can they improve their health behaviour?
- How can they improve respiratory health?
- How can RUHSA support them in maintaining positive respiratory health?
- What are the expectations from the health system (Govt. & Private) in improving respiratory health?

Is there anything you would like to add?

CLOSURE SCRIPT: To be read by researcher/group facilitator:

Thank you very much for your time and willingness to share your experiences with us today. We will include your experiences in our data to help you with better care in the future.

**In-depth interview**

(caregivers or relatives of the patients involved with patient care)

Objectives –

1. To know about the patient condition, complications from the disease and risk factors prevailing in the community
2. To identify the facilitators and barriers to treatment and follow-up

Interview Guide for in-depth interview

1. Your role in patient care
   1. How much is the patient dependant on you for medical treatment? (physically, financially or psychologically)
   2. What do you think about the patient’s health status – before and after starting treatment?
   3. Do you think continuing treatment will be beneficial for the patient?
   4. How much do you feel patient will be influenced by you for continuing treatment for his/her disease?
2. Role during complications
   1. What are the complications that have occurred or can occur due to this disease?
   2. Will you be able to identify them?
   3. What will you do when you identify such a complication in the patient?
   4. What type of support can you provide during such a situation? (physical, psychological, financial)
3. Risk factors of the disease
   1. What do you think are the risk factors of this disease?
   2. Can the risk factors be avoided?
   3. Will avoidance of risk factors help in improving the patient’s condition?
   4. What will be your role in helping her/him in avoiding risk factors?
4. Health seeking behaviour and adherence to treatment
   1. Where do you think the patient should go first when she/he develops exacerbation of symptoms or complications?
   2. Do you think the present health system (nearest Govt. hospital or RUHSA hospital) will be able to manage such a situation?
   3. What are the factors which can help the patient in continuing treatment (use medicines prescribed, use inhalers and go for regular check-up as advised)?
      1. Personal attributes of patient (self-motivation, financial ability etc.)
      2. Family support
      3. Community perception / support
      4. Health system support (FCV, Health aides, VHN, ASHA, doctors and health facilities)
      5. Any other factor
   4. What are the factors which can prevent regular use of health system and follow-up for disease?
      1. Personal attributes of patient (self-motivation, financial ability etc.)
      2. Family support
      3. Community perception / support
      4. Health system support (FCV, Health aides, VHN, ASHA, doctors and health facilities)
      5. Any other factor

**Key Informant Interview**

Block development officer, panchayat leader, health care worker, teacher, village health nurse, PHC medical officer

Objectives –

1. To know their perceptions about the capability of the existing health system to manage chronic respiratory diseases
2. To identify facilitators and barriers to CRD management

Interview Guide for Key-Informant Interview

1. Awareness of Chronic respiratory diseases in the community
   1. Are people in the community aware about chronic respiratory diseases
      1. What are the characteristics of such disease?

progressive in nature, if not treated and worsening of symptoms, leads to frequent complications

- - 1. What type of treatment and follow-up do they require? life-long treatment with medicines and inhalers, regular follow-up as prescribed
    2. Can they be diagnosed early and treated? and will it help? early diagnosis & treatment can keep the disease under control, reduce the symptoms, decrease the frequency of complications and improve respiratory health
  1. Community awareness about its risk factors
     1. Common risk factors prevalent in the community
     2. How can their exposure be prevented or minimised?
     3. What can be the role of govt. institutions / panchayat in doing so
     4. enforcing smoking bans, doing awareness campaigns, providing clean cooking fuel, open discussions in community meetings or panchayats and encourage people to stop smoking, use clean fuel and go for treatment if symptomatic

1. Perception about the role of long-term treatment in CRDs
   1. Community perception about its treatment
      1. Is the community aware what is the duration of treatment for such diseases?
      2. Does it have a supportive role to play?
      3. Will the community support educational programs?
2. Perception about use of inhalers for treatment of CRDs
3. Perception about the capability of the health system Govt. and Pvt. Including RUHSA to identify, diagnose, treat and follow-up such patients
4. Supporting factors in the community and/or health system to manage CRDs
5. Barriers/constraints in the community and/or health system to manage CRDs
