# Supplementary material for: Development of an educational intervention to reduce the burden of adult chronic lung disease in rural India: Inputs from a qualitative study
Source: PLoS One. 2021 Jul 15;16(7):e0254534. doi: 10.1371/journal.pone.0254534 (PMC8281992; doi:10.1371/journal.pone.0254534)
Supplement: S1 File — (DOCX) [file pone.0254534.s003.docx]

Complete list of Group authors of “RESPIRE Collaboration” for the manuscript:

**RESPIRE Partners –**

| **Name** | **Key affiliation** | **Role** | **Country** | **RESPIRE role** |
| --- | --- | --- | --- | --- |
| Professor Shams El Arifeen | Maternal and Child Health Division (MCHD), International Centre for Diarrhoeal Disease Research, Bangladesh (icddr,b) | Senior Director and Senior Scientist | Bangladesh | Lead: Platform III (Methodology and Data Science) |
| Professor Abdullah H. Baqui | Projahnmo Research Foundation | Scientific Adviser | Bangladesh |  |
| Professor Samir Saha | Child Health Research Foundation | Executive Director | Bangladesh | Lead: Programme 1 (Acute) |
| Dr GM Monsur Habib | Bangladesh Primary Care Respiratory Society | President | Bangladesh |  |
| Professor Rita Isaac | Rural Unit for Health and Social Affairs, Christian Medical College Vellore | Director | India |  |
| Professor Sanjay Juvekar | Vadu Rural Health Program, King Edward Memorial Hospital Research Centre (KEMHRC) Pune | Officer in charge | India | Data Access and Sharing Lead |
| Professor Ee Ming Khoo | Department of Primary Care Medicine, University of Malaya | Professor | Malaysia | Lead: Programme 2 (Chronic) |
| Associate Professor Sazlina Shariff Ghazali | Department of Family Medicine, Faculty of Medicine and Health Sciences, Universiti Putra Malaysia | Associate Professor | Malaysia |  |
| Professor Tabish Hazir | Neoventive Solutions | Principal Investigator | Pakistan | Lead: Platform II (Training and Capacity Building) |
| Dr Osman Yusuf | Allergy and Asthma Institute, Pakistan | Chief Consultant | Pakistan |  |

**RESPIRE Grantholders –**

| **Name** | **Department** | **RESPIRE role** |
| --- | --- | --- |
| Professor Aziz Sheikh | Usher Institute, The University of Edinburgh | Director |
| Professor Harry Campbell | Usher Institute, The University of Edinburgh | Co-Director \| UK lead for Pakistan |
| Professor Debby Bogaert | MRC Centre for Inflammation Research, The University of Edinburgh |  |
| Dr Kathrin Cresswell | Usher Institute, The University of Edinburgh |  |
| Professor Steve Cunningham | Child Life and Health, The University of Edinburgh |  |
| Professor David Dockrell | MRC Centre for Inflammation Research, The University of Edinburgh |  |
| Monica Fletcher | Usher Institute, The University of Edinburgh | Sustainability/scalability |
| Professor Liz Grant | Global Health Academy, The University of Edinburgh | Sustainability/scalability |
| Professor Brian McKinstry | Usher Institute. The University of Edinburgh |  |
| Professor Andrew Morris | Health Directorate, The University of Edinburgh/Scottish Government | Sustainability/scalability |
| Professor Harish Nair | Usher Institute, The University of Edinburgh | Lead: Programme I (Acute) \| UK lead for India |
| Professor John Norrie | Edinburgh Clinical Trials Unit, The University of Edinburgh | Lead: Platform III (Methodology and Data Science) |
| Dr Bright Nwaru | Usher Institute, The University of Edinburgh |  |
| Professor Hilary Pinnock | Usher Institute, The University of Edinburgh | Lead: Programme II (Chronic) \| UK lead for Malaysia |
| Professor Dave Robertson | Usher Institute, The University of Edinburgh |  |
| Professor Igor Rudan | Usher Institute, The University of Edinburgh | Lead: Platform II (Training and Capacity Building) \| UK lead for Bangladesh |
| Professor Jürgen Schwarze | Child Life and Health, The University of Edinburgh | Paediatric work overview |
| Dr Colin Simpson | Usher Institute, The University of Edinburgh |  |
| Professor Devi Sridhar | Usher Institute, The University of Edinburgh | Lead: Platform I (Stakeholder Engagement and Governance) |
| Andy Stoddart | Usher Institute, The University of Edinburgh |  |
| Professor David Weller | Usher Institute, The University of Edinburgh | Adult work overview |
| Siân Williams | International Primary Care Respiratory Group | Lead: Platform I (Stakeholder Engagement and Governance) |
| Dr Allison Worth | Edinburgh Clinical Research Facility, The University of Edinburgh |  |
| Professor Moira Whyte | MRC Centre for Inflammation Research, The University of Edinburgh |  |
| Professor Sir Alimuddin (Ali) Zumla | Division of Infection and Immunity, University College London |  |

**Research Team members –**

| Professor Aziz Sheikh | Director |
| --- | --- |
| Professor Harry Campbell | Co-Director |
| Professor Harish Nair | Lead: Programme I |
| Professor Samir Saha | Lead: Programme I |
| Professor Hilary Pinnock | Lead: Programme II |
| Professor EE Ming Khoo | Lead: Programme II |
| Professor Devi Sridhar | Lead: Platform I |
| Siân Williams | Lead: Platform I |
| Professor Igor Rudan | Lead: Platform II |
| Professor Tabish Hazir | Lead: Platform II |
| Professor John Norrie | Lead: Platform III |
| Professor Shams El Arifeen | Lead: Platform III |
| Professor Jürgen Schwarze | Paediatric Research Coordinator |
| Professor David Weller | Adult Research Coordinator |
| Monica Fletcher | Sustainability/Scalability |
| Professor Liz Grant | Sustainability/Scalability |
| Dr Sanjay K Juvekar | Data Access and Sharing Lead |
| Mohammad Shahidul Islam | 2018-19 PhD student representative |
